# Improved dialytic removal of protein-bound uraemic toxins with use of albumin binding competitors: an *in vitro* human whole blood study

**DOI:** 10.1038/srep23389

**Published:** 2016-03-22

**Authors:** Xia Tao, Stephan Thijssen, Peter Kotanko, Chih-Hu Ho, Michael Henrie, Eric Stroup, Garry Handelman

**Affiliations:** 1 University of Massachusetts Lowell, Lowell, MA, USA; 2 Renal Research Institute, NY, NY, USA; 3 Icahn School of Medicine at Mount Sinai, New York, NY, USA; 4 Fresenius Medical Care, Ogden, UT, USA

## Abstract

Protein-bound uraemic toxins (PBUTs) cause various deleterious effects in end-stage kidney disease patients, because their removal by conventional haemodialysis (HD) is severely limited by their low free fraction in plasma. Here we provide an experimental validation of the concept that the HD dialytic removal of PBUTs can be significantly increased by extracorporeal infusion of PBUT binding competitors. The binding properties of indoxyl sulfate (IS), indole-3-acetic acid (IAA) and hippuric acid (HIPA) and their binding competitors, ibuprofen (IBU), furosemide (FUR) and tryptophan (TRP) were studied in uraemic plasma. The effect of binding competitor infusion on fractional removal of PBUT was then quantified in an *ex vivo* single-pass HD model using uraemic human whole blood. The infusion of a combination of IBU and FUR increased the fractional removal of IS from 6.4 ± 0.1 to 18.3 ± 0.4%. IAA removal rose from 16.8 ± 0.3 to 34.5 ± 0.7%. TRP infusion increased the removal of IS and IAA to 10.5 ± 0.1% and 27.1 ± 0.3%, respectively. Moderate effects were observed on HIPA removal. Pre-dialyzer infusion of PBUT binding competitors into the blood stream can increase the HD removal of PBUTs. This approach can potentially be applied in current HD settings.

With better detection technologies, a broad spectrum of retained solutes in dialysis patients’ blood has been identified and characterized in recent studies^1^,^2^,^3^,^4^. This group of solutes is generally defined as uraemic toxins, although toxic effects are not established for all of these compounds. Many of these substances, most notably protein-bound uraemic toxins (PBUTs), interact negatively with biological systems^5^,^6^,^7^,^8^,^9^, and reducing the plasma levels of these compounds could improve haemodialysis (HD) outcomes^7^,^10^.

The removal of PBUTs is a major challenge for current HD technology. The removal of such solutes in conventional HD primarily relies on diffusion of the free molecules into the dialysate, which is severely limited for PBUTs due to their low free fraction and hence small diffusion gradient. For some strongly bound uraemic toxins, clearance is undetectable during a regular HD session^11^. Even for many PBUTs where there is detectable dialytic removal, the plasma level of these compounds often remains highly elevated after HD^10^,^12^,^13^,^14^.

Several new approaches have been reported in recent publications to improve the dialytic removal of PBUTs. Longer dialysis sessions^13^,^14^ and hemodiafiltration^12^,^15^ have only yielded modest improvements. Use of larger dialyzers in combination with higher dialysate flow rate (Qd) of 800 ml/min almost doubled the clearance of indoxyl sulfate (IS)^13^. Fractionated plasma separation and adsorption (FPSA) was 2 times more efficient in removing IS and p-cresol sulfate (PCS) than regular HD in a clinical study^16^, although the risk of occlusive thrombosis could be a safety concern for using FPSA for this purpose^17^.

Here we propose another innovative method for improving dialytic removal of PBUTs. Our method is based on the observation that albumin-binding ligands can influence the binding properties of albumin to other ligands through direct competition for binding sites, or by allosteric mechanisms^18^,^19^,^20^. The binding of a given ligand on albumin may alter the conformation in the vicinity of the binding sites, or directly block the diffusion path for binding of other ligands. The binding competition between different albumin ligands has been widely reported in the literature^18^,^19^,^21^,^22^,^23^,^24^,^25^. Using compounds that share the same binding sites as uraemic toxins to impede their binding is a direct approach to increase the free fraction of these uraemic toxins. By infusing binding competitors (displacers) upstream of the dialyzer into the blood compartment, the diffusion gradients and the dialytic removal of PBUTs will be increased. This has been demonstrated *in vitro* using human serum albumin solution^26^. The purpose of this study was to provide experimental validation of the PBUT displacement approach using human whole blood in an *ex vivo* dialysis model, and to determine whether the presence of red blood cells or endogenous albumin ligands in human plasma has any appreciable impact on the effect of PBUT displacement in comparison to previous *in vitro* studies with human serum albumin.

## Results

### PBUT displacement in uraemic plasma in a static model system

**Figure 1:**
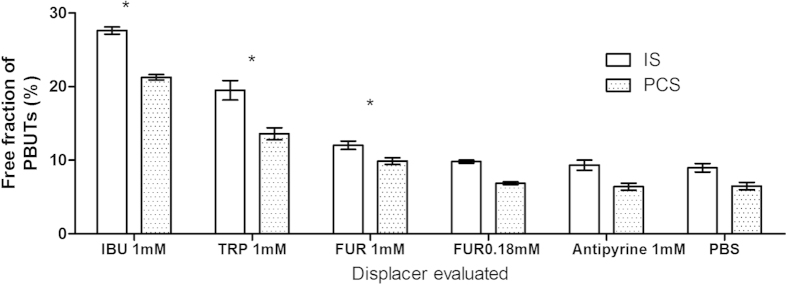
IS and PCS displacement in uraemic plasma by furosemide, tryptophan and ibuprofen, determined in static RED assays. Displacer concentration was 1 mmol/l, unless otherwise indicated. IS: indoxyl sulfate; PCS: p-cresol sulfate; IBU: ibuprofen; TRP: tryptophan; FUR: furosemide; PBS: phosphate buffered saline. Bars denote mean, error bars denote standard error of the mean (SEM), N = 3. *P < 0.05, compared to PBS.

**Figure 2:**
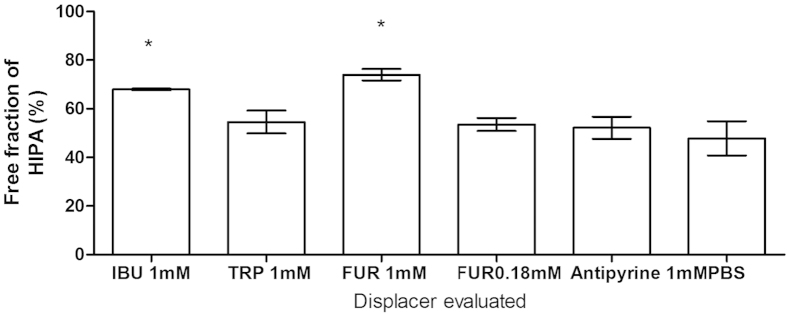
HIPA displacement in uraemic plasma by furosemide, tryptophan and ibuprofen, determined in static RED assays. Displacer concentration was 1 mmol/l, unless otherwise indicated. IBU: ibuprofen; TRP: tryptophan; FUR: furosemide; PBS: phosphate buffered saline. Mean ± SEM, N = 3. *P < 0.05, compared to PBS.

Ibuprofen, which possesses the highest binding affinity among the displacers tested in the study, increased the free fraction of both IS and PCS approximately 3-fold in uraemic plasma, higher than the free fraction generated by tryptophan (about a 2-fold increase) and furosemide (about a 1.3-fold increase) (Fig. 1). Addition of antipyrine (a negative control), which lacks protein binding capability^27^,^28^, did not lead to a significant change in uraemic toxin protein binding (Figs 1 and 2).

L-Tryptophan shares the same primary site as IS and PCS, while furosemide shares the same primary binding site as hippuric acid (HIPA). Thus, tryptophan displaces IS and PCS more efficiently than HIPA (Fig. 1). In contrast, furosemide, which had limited displacement effect on IS and PCS, showed significant displacement of HIPA (Fig. 2).

### Dose-response relationship for indoxyl sulfate displacement by ibuprofen, and synergistic effect of ibuprofen and furosemide in normal human plasma in a static dialysis setup

**Figure 3:**
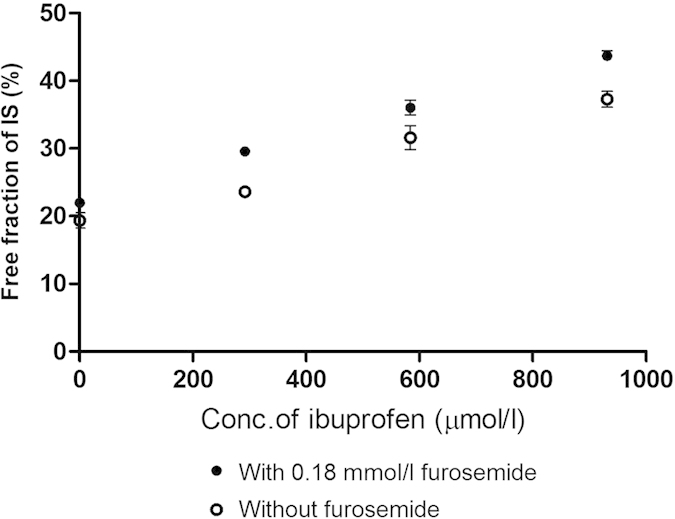
Dose-response relationships for IS displacement by ibuprofen, and synergistic effect of ibuprofen and furosemide in normal human plasma, determined in static RED assays. Solid circle: ibuprofen with 180 μmol/l furosemide added; open circle: ibuprofen alone. Mean ± SEM, N = 3.

While furosemide alone at a concentration of 0.18 mmol/l did not induce a significant increase in the free fraction of IS (Fig. 1), the same concentration of furosemide was able to boost the IS displacement effect of ibuprofen when given simultaneously, suggesting additive or synergistic effects of these two binding competitors (Fig. 3). The range of ibuprofen concentrations tested in these studies was established based on the plasma level of ibuprofen that could occur with clinically relevant intravenous administration as described in the method section.

### Effect of displacer infusion on PBUT removal from whole blood in a single-pass haemodialysis model

**Figure 4:**
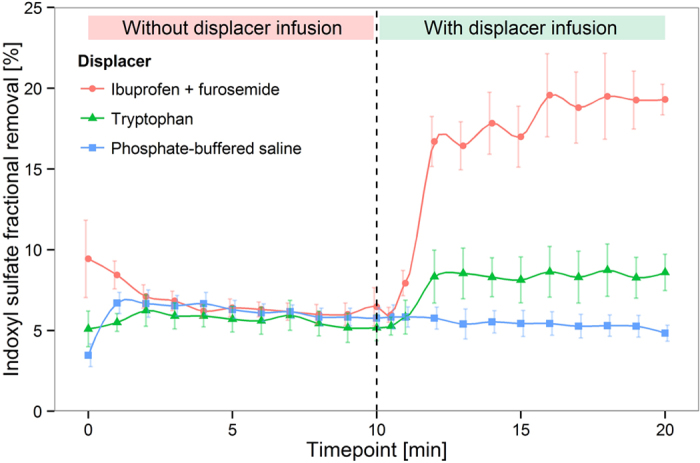
Indoxyl sulfate displacement in human whole blood HD model. Mean ± SEM, N = 3.

**Figure 5:**
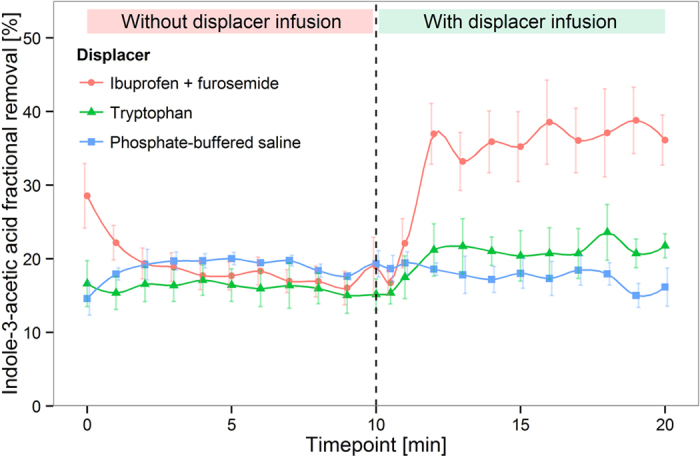
Indoleacetic acid displacement in human whole blood HD model. Mean ± SEM, N = 3.

**Figure 6:**
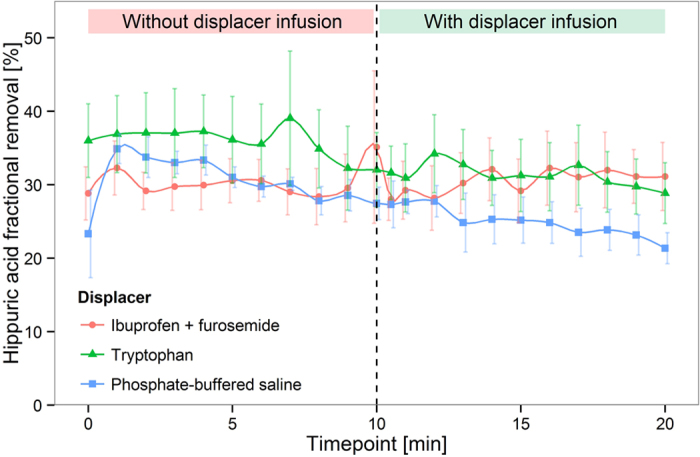
Hippuric acid displacement in human whole blood HD model. Mean ± SEM, N = 3.

**Figure 7:**
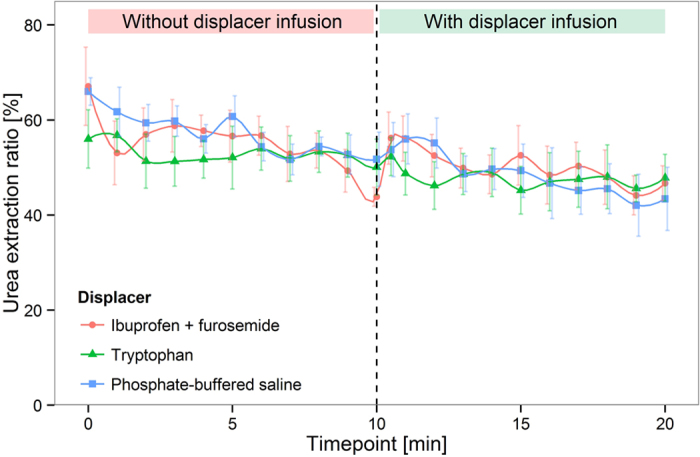
Urea extraction ratio in human whole blood HD model. Mean ± SEM, N = 3.

Figures 4 and 5 show that a 2.9-fold increase of IS removal and a 2.1-fold increase of indoleactic acid (IAA) removal were achieved by infusion of ibuprofen and furosemide in an *in vitro* HD model (P < 0.0001). Tryptophan infusion was able to increase the removal of IS and IAA by a factor of 1.4 (P < 0.0001) and 1.3 (P < 0.0001), respectively. HIPA removal, on the other hand, was only slightly increased by infusion of the ibuprofen/furosemide combination, and virtually unaffected by infusion of tryptophan (Fig. 6). Likewise, the urea extraction ratio was unaffected by infusion of binding competitors or phosphate buffered saline (PBS) (Fig. 7). Infusion of PBS as a control did not lead to an increase in removal of any of the PBUTs.

## Discussion

To date, more than 90 substances have been reported to accumulate in patients with advanced renal failure^1^,^29^. Although the toxicity of many of these substances remains undefined, high levels of IS and PCS have been demonstrated to be associated with damage to multiple systems, such as the kidney^5^,^8^,^9^,^30^, cardiovascular system^7^,^30^,^31^,^32^, smooth muscle^33^,^34^ and erythrocytes^35^. AST-120, an oral carbon adsorbent, decreases the plasma levels of colon-derived PBUTs by inhibiting the intestinal absorption of their metabolism precursors, and may slow the rate of loss of renal function, and delay the progression of cardiovascular damage in chronic kidney disease^31^,^36^,^37^. However, overall improvement of dialysis patients’ clinical outcomes may require a broader strategy to increase PBUT removal that goes beyond just the colon-derived PBUTs. Increasing the removal of PBUTs during HD could offer an opportunity to significantly improve the poor clinical outcomes and low quality of life that are so prevalent in dialysis patients. This study offers proof of concept that PBUT removal during HD can be substantially increased by infusion of binding competitors upstream of the dialyzer. The approach described here lends itself in principle to routine application during HD. However, we are not advocating the routine application of the displacers used in this proof-of-principle study.

The results from this study are in full agreement with the established knowledge of albumin-ligand binding mechanisms. Albumin is known to be an allosteric protein which changes its conformation following ligand binding^20^,^38^,^39^,^40^,^41^. Interdependent alterations of albumin binding properties caused by the binding of uraemic toxins, endogenous substances and drugs have been established^42^,^43^,^44^,^45^,^46^,^47^. PBUTs vary in their binding sites and binding affinities. Sudlow’s site I and II on human albumin have been reported to be the primary binding sites for some protein bound uraemic toxins^18^,^19^,^20^,^43^,^44^,^45^,^47^,^48^. Within the large family of PBUTs, we chose to study IS as an example of a strongly albumin bound toxin^18^,^19^,^44^,^45^, IAA as a toxin with medium binding affinity towards albumin^44^,^48^,^49^, and HIPA as an example of a weakly albumin bound toxin^43^,^44^,^45^. We selected model displacers based on their binding characteristics reported in the literature^20^,^24^,^50^,^51^,^52^,^53^,^54^,^55^,^56^,^57^, and on data that we collected from our preliminary studies in a static displacement model. Ibuprofen was selected as an example to demonstrate method efficiency with a high affinity displacer, while tryptophan was selected as a low affinity displacer. Furosemide was used to show the effect of a combination of both site I and site II displacers. Feasibility for a proof-of-concept study in humans is another reason we considered when choosing these displacers. Our data demonstrate that strongly bound displacers alter the binding of uraemic toxins on albumin more significantly and result in a higher free fraction of the toxins than displacers with lower albumin binding affinity. It is possible that when the binding of IS at its primary binding site is impeded by site II displacers, its secondary binding site may still be accessible. Thus, a combination of displacers targeting different binding sites can further increase the removal of a given uraemic toxin, as demonstrated in this study. There are conflicting reports in the literature on the primary binding site of HIPA^43^,^44^,^45^. Our data indicate the primary binding site of HIPA is probably located at Sudlow site I, because the site I displacer furosemide demonstrated greater HIPA displacement from albumin than ibuprofen and tryptophan, whose primary albumin binding site is Sudlow site II.

Considering future clinical applications, we designed our HD model to be compatible with the clinical setup used in the HD clinic. In a clinical HD setting, an infusion pump could be connected between the cannula or catheter and the arterial line. In typical haemodialysis systems, the arterial segment of the blood tubing has a filling volume of about 100 ml, allowing approximately 15 seconds of transit time between the displacer infusion site and the dialyzer at a Qb of 400 ml/min. In our experiments, we connected the displacer infusion upstream of the dialyzer, allowing for 15 seconds of transit time between the infusion site and the dialyzer. Our results show that this time interval is sufficient for the binding and displacement reactions to occur.

We also designed the study to achieve clinically reasonable plasma concentrations of ibuprofen and furosemide. Both of these drugs are commercially available as formulations for intravenous infusion. The concentrations of ibuprofen and furosemide that were targeted in our study are similar to what might be observed clinically with dosing protocols for human subjects consistent with approved rates of infusion for these compounds.

We successfully demonstrated the feasibility of a novel potential therapeutic method of enhancing the removal of highly protein-bound uraemic toxins in HD with the use of displacers. Non-protein-bound substances are not affected by the described displacement method. Of note, we are not advocating the routine clinical use of these particular displacers in a clinical setting. They serve merely as model compounds to demonstrate the concept of displacement therapy for lowering PBUT levels during HD. The primary purpose of this study was to demonstrate the feasibility and efficacy of the PBUT displacement approach in a setting comparable to the one encountered in clinical routine HD. Future clinical pilot trials will be required to document the *in vivo* efficacy of this approach in a clinical HD setting with human subjects. Such trials will also serve to generate important *in vivo* data on PBUT and displacer kinetics for use in mathematical models. Future efforts will also have to focus on identification of suitable displacer substances that are both efficacious and safe for long-term routine clinical use in patients during HD. Our study shows that endogenous molecules, nutritional supplements and high affinity pharmaceutical compounds can all potentially be enlisted in the screening pool for suitable displacer candidates. A displacer molecule should ideally be biologically inert (if not salutary) when used in dialysis patients, as well as effective at displacing PBUTs. The ideal displacer would induce a significant increase in PBUT removal during HD, and yet be rapidly metabolized into biologically inert compounds that are readily removed by HD or eliminated via biliary excretion.

## Methods

### Materials

Rapid Equilibrium Dialysis (RED) cartridges (8,000 Da molecular weight cut-off) were purchased from Pierce Thermo Fisher (Rockford, IL, USA). Acetonitrile (HPLC grade) was obtained from Fisher Chemical (Thermo Fisher, USA). Furosemide, IAA, HIPA, IS, indole, tryptophan, p-cresol and PBS were obtained from Sigma (St Louis, MO, USA). PCS was synthesized following the protocol of Feigenbaum and Neuberg^58^. Ibuprofen solution (Caldolor injection 100 mg/ml) was obtained from Cumberland Pharmaceuticals Inc. (Nashville, TN, USA). Furosemide was dissolved in 0.05 mol/l sodium hydroxide, and then mixed with Caldolor. The final concentration of ibuprofen was 117 mmol/l and furosemide was 23 mmol/l in the mixture, with a pH of approx. 8.4. Tryptophan infusion solution was prepared by dissolving tryptophan powder in PBS with a final concentration of 50 mmol/l. Acid concentrate (Naturalyte, cat#08-4225-1) and sodium bicarbonate concentrate (Naturalyte sodium bicarbonate concentrate, cat#08-4000-LB) were obtained from Fresenius Medical Care North America (FMCNA, Waltham, MA, USA), and prepared to make complete dialysate, following the manufacturer’s instructions. The final pH was adjusted to 7.4. The Envoy 500 clinical analyzer was purchased from Vital Diagnostics (RI, USA). Urea Infinity reagent for Envoy was purchased from Thermo Fisher (Rockford, IL, USA)

Normal human plasma with heparin (from a six-donor pool) was purchased from Bioreclamation (NY, USA, lot# BRH739237). Twenty-four heparinized uraemic plasma samples from 18 HD patients (obtained from a de-identified bio-repository) were pooled to create a uraemic plasma pool. The pooled uraemic plasma contained endogenous (mean ± SEM, 3 measurements) 23.6 ± 5.6 μmol/l tryptophan, 181.9 ± 6.2 μmol/l IS, 326.5 ± 74.1 μmol/l HIPA and also PCS at a level not quantitated. De-identified human whole blood and informed consent were obtained in cooperation with a local blood bank from healthy volunteer donors (approved by the Ogden Regional Medical Center Institutional Review Board; IRB number 495825-1) on the morning of the experiments. The whole blood was heparinized with 15 IU/ml unfractionated heparin (150,000 USP/l).

All experiments were performed in accordance with relevant guidelines and regulations.

Dialysis experiments were performed using miniature polysulfone dialyzers produced by the Biotechnology Research Group, Dialyzer R&D, of FMCNA in Ogden, Utah, USA. The specifications of the dialyzers are 0.039 m^2^ surface area with the same pore size distribution as Optiflux^®^ F160NR fibers and 15.5 cm fiber length. Peristaltic pumps (Watson-Marlow 120U) were obtained from Watson-Marlow Fluid Technology Group North America (Wilmington, MA, USA). A syringe pump for the displacer infusion was purchased from Kd Scientific Inc. (Holliston, MA, USA). Plasma and dialysate samples were collected using the Sarstedt S-Monovette^®^ Blood Collection System (EDTA) (Sarstedt AG & Co, Nümbrecht Germany).

### Studies of the effect of binding competitors on the albumin binding of PBUTs in uraemic plasma with static dialysis

We evaluated the albumin binding of uraemic toxins and their interaction with displacers in human uraemic plasma utilizing the RED device. Candidate displacers were mixed with uraemic plasma before incubation. The test mixture (300 μl) was added into the sample chamber, while the buffer chamber was filled with 500 μl PBS. After 4 hours of incubation (37 °C, 250 rpm), samples were collected from both chambers for HPLC analysis. The concentration of all displacers and negative control (antipyrine) in the incubations was 1 mmol/l. For furosemide, incubations at final concentration of 0.18 mmol/l were also included since this is the clinically relevant concentration for intravenous administration.

### Studies of dose-response effect of ibuprofen and synergistic effect of furosemide and ibuprofen on PBUT displacement in normal human plasma with static dialysis

Normal plasma was spiked with IS (150 μmol/l) and mixed with ibuprofen over a concentration range from 0 to 931 μmol/l to generate a dose-dependent displacement curve using the RED device. Further experiments were performed with the addition of furosemide (final concentration 182 μmol/l), to test the hypothesis that addition of furosemide to ibuprofen would produce greater displacement of IS.

Ibuprofen and furosemide doses were calculated to achieve a range of plasma concentrations that one might encounter when administering these drugs into the extracorporeal circuit following clinical patient-dosing protocols. Infusion of 800 mg ibuprofen into the arterial line over 30 min, with a Qb of 200 ml/min and a hematocrit of approximately 31%, would result in a plasma concentration of approximately 931 μmol/l . Infusion of 250 mg furosemide over 30 min in this setting would results in a plasma concentration of 182 μmol/l.

### Studies of PBUT removal with a haemodialysis model

Dialysis experiments were conducted with a volume of 300 ml of heparinized human whole blood spiked with uraemic toxins, referred to as uraemic blood in the study. IS, IAA and HIPA were added to achieve final plasma concentrations of 150 μmol/l, 15 μmol/l and 400 μmol/l, respectively, except in the experiments with tryptophan as a displacer, where a HIPA concentration of 1 mmol/l was used.

Experiments were conducted at 37 °C, with a Qb of 12.5 ml/min and counter-current dialysate flow at a Qd of 25 ml/min. Blood and dialysate were perfused through each compartment in single-pass. An infusion pump was connected between the blood pump and dialyzer blood inlet. The tube segment between the infusion site and the dialyzer blood inlet was 45 cm long, with a 3 mm inner diameter, allowing a 15-second transit time between displacer infusion site and dialyzer inlet.

**Figure 8:**
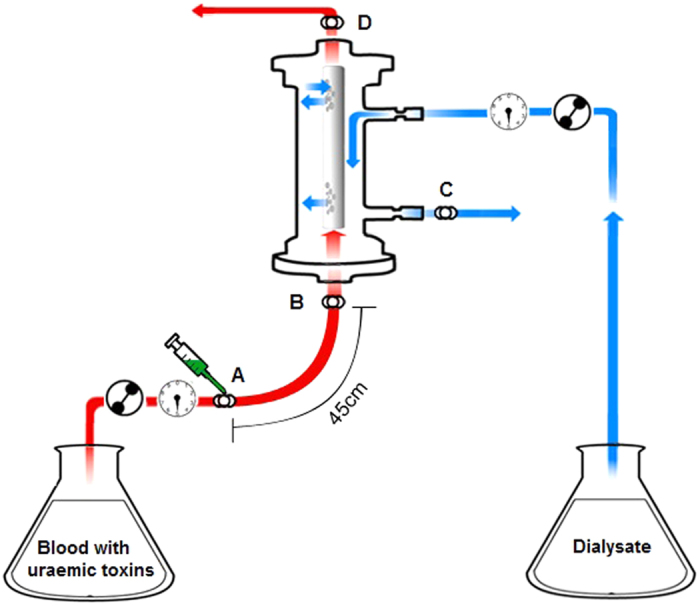
Setup of *in vitro* dialysis model using human whole blood. (**A**) infusion site of displacers. (**B**) sample collection site for blood inlet. (**C**) sample collection site for dialysate outlet. (**D**) sample collection site for blood outlet.

The system was primed prior to each experiment, initially with saline, and then with “blank” whole blood without uraemic toxins in the blood circuit, and with dialysate in the dialysate circuit. With the blank blood in recirculation, we manually adjusted the trans-membrane pressure between two circuits so there was zero net ultrafiltration at the beginning of the experiment. Blank whole blood in the circuit was replaced with uraemic blood at the start of the experiments. Details of the experimental model setup are illustrated as in Fig. 8. All experiments were carried out in triplicate.

Human whole blood containing uraemic toxins was circulated for 10 minutes without displacer to measure the baseline uraemic toxin single-pass removal. At 10 min, tryptophan, ibuprofen/furosemide, or PBS were infused into the blood line. The target level of tryptophan was 1 mmol/l in plasma, and 650 μmol/l ibuprofen and 129 μmol/l furosemide in whole blood. The infusion concentrations of ibuprofen and furosemide were determined according to the manufacturer’s prescription recommendation as described above. The infusion rate was 70 μl/min for ibuprofen (116 mmol/l) /furosemide (23 mmol/l), 138–150 μl/min for tryptophan (50 mmol/l), and 146 μl/min for PBS.

One milliliter samples for analysis were collected from the dialyzer blood inlet, blood outlet and dialysate outlet at 1-minute intervals throughout the experiment.

### Sample analysis for protein-bound uraemic toxins and urea

Blood samples were centrifuged at 13200 rpm for 3 mins. The plasma layer was collected and stored at −80 °C. Plasma samples (50 μl) were precipitated with 100 μl of ice-cold acetonitrile to precipitate the proteins. The samples were then vortexed thoroughly and centrifuged at 3000 rpm for 10 minutes. The supernatant, containing the uraemic toxins and displacers, was collected and further diluted 1:1 with deionized water before HPLC injection. The dialysate samples (100 μl) were diluted with 100 μl water before HPLC injection.

Measurements were calibrated with standard curves for all analytes, and were linear over the concentration range of the experiments. Measurements were performed on an Agilent 1100 system (Agilent Technologies, Dover, DE, USA) equipped with a C18 column (Kinetex 5 μm C18 100 Å, LC Column 150 × 4.6 mm, Phenomenex, Torrance, CA, USA). Mobile phase A: ammonium formate, 20 mmol/l, pH 4. Mobile phase B: acetonitrile. The gradient profile for mobile phase B was: 0–2 min, 15%; 11–14 min, 100%; 14.2–16 min, 15%; and the flow rate gradient was 0–11.2 min, 0.4 ml/min; 11.2–16 min 0.5 ml/min. The fluorescent detector was programmed with the following timetable: 0–9 min, excitation at 280 nm and emission at 360 nm, for detection of tryptophan and IS; 9–10.5 min, excitation at 214 nm and emission at 309 nm for PCS; and then for the remainder of the HPLC run, excitation at 280 nm and emission at 360 nm, for detection of IAA and ibuprofen. HIPA and furosemide were detected at 230 nm with the UV-Vis detector. The injection volume was 5 μl.

Urea was measured on Envoy clinical biochemical analyzer using Thermo Fisher Infinity urea liquid stable reagent.

### Calculation of removal of uraemic toxins and urea from whole blood

PBUT removal from whole blood was expressed as fractional removal, calculated as the amount per unit of time leaving the dialysate outlet, as a percentage of the amount per unit of time entering at the blood inlet, in accordance with


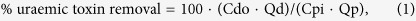


where Cpi and Cdo are the concentrations of each uraemic toxin in the plasma inlet and dialysate outlet streams, respectively. Qd is the dialysate flow rate, and Qp is the plasma flow rate calculated from blood flow rate and hematocrit according to Qp = Qb·(1-Hct). Urea removal was specified as the single pass extraction ratio (ER) in percent, calculated as


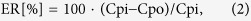


where Cpi and Cpo are the plasma concentrations in the blood inlet and blood outlet streams, respectively.

### Statistics

For PBUT displacement in RED assays, Dunnett’s post-analysis comparisons to PBS were performed by one way ANOVA using GraphPad Prism (GraphPad Software, La Jolla, CA).

For the PBUT displacement experiments with the HD model, data were converted into the percent change during the intervention phase, compared to the baseline phase for that experiment. After pairing similar observations in the two phases for an individual experiment (minute by minute, pairing baseline phase with intervention phase) and calculating the paired differences, the value for each observation was divided by the corresponding paired difference SD. This transformation created independent observations with theoretically equal variances (within patients and between patients) for the paired differences under the null hypothesis. Each paired t test was two-tailed; a P value less than 0.05 was taken as statistically significant. The data from the first 2 minutes of each phase (baseline and intervention) were excluded to restrict the analysis to data with stable values during that phase. Calculations were done with SPSS 16.0 on an IBM-PC.

## Additional Information

**How to cite this article**: Tao, X. *et al.* Improved dialytic removal of protein-bound uraemic toxins with use of albumin binding competitors: an *in vitro* human whole blood study. *Sci. Rep.* **6**, 23389; doi: 10.1038/srep23389 (2016).

## References

[b1] Vanholder R. *et al.* Review on uremic toxins: Classification, concentration, and interindividual variability. Kidney Int. 63, 1934–1943 (2003). 12675874 10.1046/j.1523-1755.2003.00924.x

[b2] Liabeuf S., Drüeke T. B. & Massy Z. a. Protein-bound uremic toxins: New insight from clinical studies. Toxins (Basel). 3, 911–919 (2011). 22069747 10.3390/toxins3070911 PMC3202851

[b3] Vanholder R., Glorieux G., De Smet R. & Lameire N. New insights in uremic toxins. Kidney Int. Suppl. 63, S6–S10 (2003). 12694297 10.1046/j.1523-1755.63.s84.43.x

[b4] Meijers B. K. I. *et al.* p-Cresyl sulfate and indoxyl sulfate in hemodialysis patients. Clin. J. Am. Soc. Nephrol. 4, 1932–8 (2009). 19833905 10.2215/CJN.02940509 PMC2798868

[b5] Sun C.-Y., Chang S.-C. & Wu M.-S. Uremic toxins induce kidney fibrosis by activating intrarenal renin-angiotensin-aldosterone system associated epithelial-to-mesenchymal transition. PLoS One 7, e34026 (2012). 22479508 10.1371/journal.pone.0034026 PMC3316590

[b6] Vanholder R., Schepers E., Pletinck A., Nagler E. V. & Glorieux G. The uremic toxicity of indoxyl sulfate and p-cresyl sulfate: a systematic review. J. Am. Soc. Nephrol. 25, 1897–907 (2014). 24812165 10.1681/ASN.2013101062 PMC4147984

[b7] Barreto F. C. *et al.* Serum indoxyl sulfate is associated with vascular disease and mortality in chronic kidney disease patients. Clin. J. Am. Soc. Nephrol. 4, 1551–8 (2009). 19696217 10.2215/CJN.03980609 PMC2758258

[b8] Watanabe H. *et al.* p-Cresyl sulfate causes renal tubular cell damage by inducing oxidative stress by activation of NADPH oxidase. Kidney Int. 83, 582–92 (2013). 23325087 10.1038/ki.2012.448

[b9] Saito S. *et al.* Indoxyl sulfate-induced activation of (pro)renin receptor is involved in expression of TGF-β1 and α-smooth muscle actin in proximal tubular cells. Endocrinology 155, 1899–907 (2014). 24601883 10.1210/en.2013-1937

[b10] Marquez I. O. *et al.* Contribution of residual function to removal of protein-bound solutes in hemodialysis. Clin. J. Am. Soc. Nephrol. 6, 290–6 (2011). 21030575 10.2215/CJN.06100710 PMC3052218

[b11] Lesaffer G., De Smet R., Lameire N., Dhondt A., Duym P. & Vanholder R. Intradialytic removal of protein-bound uraemic toxins: role of solute characteristics and of dialyser membrane. Nephrol Dial Transpl. 15, 50–57 (2000). 10.1093/ndt/15.1.50 10607767

[b12] Krieter D. H. *et al.* Protein-bound uraemic toxin removal in haemodialysis and post-dilution haemodiafiltration. Nephrol. Dial. Transplant. 25, 212–8 (2010). 19755476 10.1093/ndt/gfp437

[b13] Sirich T. L., Luo F. J. G., Plummer N. S., Hostetter T. H. & Meyer T. W. Selectively increasing the clearance of protein-bound uremic solutes. Nephrol. Dial. Transplant. 27, 1574–1579 (2012). 22231033 10.1093/ndt/gfr691 PMC3315673

[b14] Basile C. *et al.* Removal of uraemic retention solutes in standard bicarbonate haemodialysis and long-hour slow-flow bicarbonate haemodialysis. Nephrol. Dial. Transplant. 26, 1296–1303 (2011). 20813765 10.1093/ndt/gfq543

[b15] Meert N. *et al.* Effective removal of protein-bound uraemic solutes by different convective strategies: A prospective trial. Nephrol. Dial. Transplant. 24, 562–570 (2009). 18809977 10.1093/ndt/gfn522

[b16] Brettschneider F. *et al.* Removal of Protein-Bound, Hydrophobic Uremic Toxins by a Combined Fractionated Plasma Separation and Adsorption Technique. Artif. Organs 37, 409–416 (2013). 23330821 10.1111/j.1525-1594.2012.01570.x

[b17] Meijers B. K. *et al.* Removal of the Uremic Retention Solute p -Cresol Using Fractionated Plasma Separation and Adsorption. Artif. Organs 32, 214–219 (2007). 18201285 10.1111/j.1525-1594.2007.00525.x

[b18] Sakai T. *et al.* Interaction mechanism between indoxyl sulfate, a typical uremic toxin bound to site II, and ligands bound to site I of human serum albumin. Pharm. Res. 18, 520–524 (2001). 11451040 10.1023/a:1011014629551

[b19] Watanabe H. *et al.* Interaction between two sulfate-conjugated uremic toxins, p-cresyl sulfate and indoxyl sulfate, during binding with human serum albumin. Drug Metab. Dispos. 40, 1423–1428 (2012). 22513409 10.1124/dmd.112.045617

[b20] Fanali G. *et al.* Human serum albumin: From bench to bedside. Mol. Aspects Med. 33, 209–290 (2012). 22230555 10.1016/j.mam.2011.12.002

[b21] Acta B., Chemistry P., Utrecht G. H. & Utrecht G. E. The role of albumin conformation in the binding of diazepam to human serum albumin. 6, 2–9 (1980). 10.1016/0005-2795(80)90123-3 7213648

[b22] Sudlow G., Birkett D. J. & Wade D. N. Further characterization of specific drug binding sites on human serum albumin. Mol. Pharmacol. 12, 1052–1061 (1976). 1004490

[b23] Sudlow G., Birkett D. J. & Wade D. N. Characterization of two specific drug binding sites on human serum albumin. Mol. Pharmacol. 11, 824–832 (1975). 1207674

[b24] Kansy M., Gerber P. R., Kratochwil N. a, Huber W. & Mu F. Predicting plasma protein binding of drugs: a new approach. Biochem. Pharmacol. 64, 1355–1374 (2002). 12392818 10.1016/s0006-2952(02)01074-2

[b25] Grudniewska A., Gniłka R. & Wawrzeńczyk C. Ligand Binding Strategies of Human Serum Albumin:How Can the Cargo be Utilized? Chirality 22, 929–935 (2010). 19319989 10.1002/chir.20709

[b26] Tao X., Thijssen S., Levin N., Kotanko P. & Handelman G. Enhanced Indoxyl Sulfate Dialyzer Clearance with the Use of Binding Competitors. Blood Purif. 39, 323–330 (2015). 25998324 10.1159/000381008

[b27] Fraser H. S., Mucklow J. C., Murray S. & Davies D. S. Assessment of antipyrine kinetics by measurement in saliva. Br. J. Clin. Pharmacol. 3, 321–325 (1976). 973966 10.1111/j.1365-2125.1976.tb00610.x PMC1428876

[b28] Mehta M. U. *et al.* Antipyrine kinetics in liver disease and liver transplantation. Clin. Pharmacol. Ther. 39, 372–377 (1986). 3514052 10.1038/clpt.1986.57 PMC2954767

[b29] Duranton F. *et al.* Normal and pathologic concentrations of uremic toxins. J. Am. Soc. Nephrol. 23, 1258–70 (2012). 22626821 10.1681/ASN.2011121175 PMC3380651

[b30] Yu M., Kim Y. J. & Kang D.-H. Indoxyl sulfate-induced endothelial dysfunction in patients with chronic kidney disease via an induction of oxidative stress. Clin. J. Am. Soc. Nephrol. 6, 30–9 (2011). 20876676 10.2215/CJN.05340610 PMC3022246

[b31] Fujii H. *et al.* Oral charcoal adsorbent (AST-120) prevents progression of cardiac damage in chronic kidney disease through suppression of oxidative stress. Nephrol. Dial. Transplant 24, 2089–95 (2009). 19188341 10.1093/ndt/gfp007

[b32] Lekawanvijit S. *et al.* Does indoxyl sulfate, a uraemic toxin, have direct effects on cardiac fibroblasts and myocytes? Eur. Heart J. 31, 1771–9 (2010). 20047993 10.1093/eurheartj/ehp574

[b33] Muteliefu G., Enomoto A., Jiang P., Takahashi M. & Niwa T. Indoxyl sulphate induces oxidative stress and the expression of osteoblast-specific proteins in vascular smooth muscle cells. Nephrol. Dial. Transplant 24, 2051–8 (2009). 19164326 10.1093/ndt/gfn757

[b34] Chitalia V. C. *et al.* Uremic serum and solutes increase post-vascular interventional thrombotic risk through altered stability of smooth muscle cell tissue factor. Circulation 127, 365–376 (2013). 23269489 10.1161/CIRCULATIONAHA.112.118174 PMC4407990

[b35] Ahmed M. S., Abed M., Voelkl J. & Lang F. Triggering of suicidal erythrocyte death by uremic toxin indoxyl sulfate. BMC Nephrol. 14, 244; doi: 10.3390/toxins6010054 (2013). 24188099 PMC4228285

[b36] Schulman G., Vanholder R. & Niwa T. AST-120 for the management of progression of chronic kidney disease. Int. J. Nephrol. Renovasc. Dis. 7, 49–56 (2014). 24501542 10.2147/IJNRD.S41339 PMC3912158

[b37] Hatakeyama S. *et al.* Effect of an oral adsorbent, AST-120, on dialysis initiation and survival in patients with chronic kidney disease. Int. J. Nephrol. 2012, 376128; doi: 10.1155/2012/376128 (2012). 22288014 PMC3263620

[b38] Ascenzi P. & Fasano M. Serum heme-albumin: An allosteric protein. IUBMB Life 61, 1118–1122 (2009). 19946891 10.1002/iub.263

[b39] Ascenzi P. *et al.* Allosteric modulation of drug binding to human serum albumin. Mini Rev. Med. Chem. 6, 483–489 (2006). 16613585 10.2174/138955706776361448

[b40] Bojko B. *et al.* Alterations of furosemide binding to serum albumin induced by increased level of fatty acid. J. Pharm. Biomed. Anal. 51, 273–277 (2010). 19709838 10.1016/j.jpba.2009.07.025

[b41] Zenei T. & Hiroshi T. Specific and non-specific ligand binding to serum albumin. Biochem. Pharmacol. 34, 1999–2005 (1985). 10.1016/0006-2952(85)90322-3 4004916

[b42] Takamura N., Maruyama T. & Otagiri M. Effects of urernic toxins and fatty acids on serum protein binding of furosemide: Possible mechanism of the binding defect in uremia. Clin. Chem. 43, 2274–2280 (1997). 9439444

[b43] Zaidi N., Ajmal M. R., Rabbani G., Ahmad E. & Khan R. H. A Comprehensive insight into binding of hippuric acid to human serum albumin: A study to uncover its impaired elimination through hemodialysis. PLoS One 8, e71422; doi: 10.1371/journal.pone.0071422 (2013). 23951159 PMC3739763

[b44] Sakai T., Takadate A. & Otagiri M. Characterization of binding site of uremic toxins on human serum albumin. Biol. Pharm. Bull. 18, 1755–1761 (1995). 8787801 10.1248/bpb.18.1755

[b45] Sarnatskaya V. V. *et al.* Effect of protein-bound uraemic toxins on the thermodynamic characteristics of human albumin. Biochem. Pharmacol. 63, 1287–1296 (2002). 10.1016/s0006-2952(02)00869-9 11960605

[b46] Zaidi N. *et al.* Biophysical insight into furosemide binding to human serum albumin: A study to unveil its impaired albumin binding in uremia. J. Phys. Chem. B 117, 2595–2604 (2013). 10.1021/jp3069877 23438181

[b47] Meijers B. K. I., Bammens B., Verbeke K. & Evenepoel P. A Review of Albumin Binding in CKD. Am. J. Kidney Dis. 51, 839–850 (2008). 18436096 10.1053/j.ajkd.2007.12.035

[b48] Šoškić M. & Magnus V. Binding of ring-substituted indole-3-acetic acids to human serum albumin. Bioorganic Med. Chem. 15, 4595–4600 (2007). 10.1016/j.bmc.2007.04.005 17481907

[b49] Conrad M. L., Moser A. C. & Hage D. S. Evaluation of indole-based probes for high-throughput screening of drug binding to human serum albumin: analysis by high-performance. J Sep Sci . 32, 1145–1155 (2009). 19296478 10.1002/jssc.200800567 PMC2766535

[b50] Di Masi A. *et al.* Ibuprofen binding to secondary sites allosterically modulates the spectroscopic and catalytic properties of human serum heme-albumin. FEBS J. 278, 654–662 (2011). 21205199 10.1111/j.1742-4658.2010.07986.x

[b51] Cheruvallath V. K., Riley C. M., Narayanan S. R., Lindenbaum S. & Perrin J. H. A quantitative circular dichroic investigation of the binding of the enantiomers of ibuprofen and naproxen to human serum albumin. J. Pharm. Biomed. Anal. 15, 1719–1724 (1997). 9260668 10.1016/s0731-7085(96)01956-5

[b52] Yamasaki K., Chuang V. T. G., Maruyama T. & Otagiri M. Albumin-drug interaction and its clinical implication. Biochim. Biophys. Acta-Gen. Subj. 1830, 5435–5443 (2013). 10.1016/j.bbagen.2013.05.005 23665585

[b53] McMenamy R. H. & Oncley J. L. The specific binding of L-tryptophan to serum albumin. J. Biol. Chem. 233, 1436–1447 (1958). 13610854

[b54] Cunningham V. J., Hay L. & Stoner H. B. The binding of L-tryptophan to serum albumins in the presence of non-esterified fatty acids. Biochem J 146, 653–658 (1975). 1147909 10.1042/bj1460653 PMC1165355

[b55] Kragh-Hansen U. Relations between high-affinity binding sites for L-tryptophan, diazepam, salicylate and Phenol Red on human serum albumin. Biochem. J. 209, 135–142 (1983). 6847607 10.1042/bj2090135 PMC1154064

[b56] Takamura N., Maruyama T. & Otagiri M. Effects of urernic toxins and fatty acids on serum protein binding of furosemide: Possible mechanism of the binding defect in uremia. Clin. Chem. 43, 2274–2280 (1997). 9439444

[b57] Viani A., Cappiello M., Silvestri D. & Pacifici G. M. Binding of furosemide to albumin isolated from human fetal and adult serum. Dev. Pharmacol. Ther. 16, 33–40 (1991). 1879250

[b58] Feigenbaum J. & Neuberg C. A. Simplified Method for the preparation of aromatic sulfuric acid esters. Notes 63, 3529–3530 (1941).

